# Nutritional and behavioral determinants of adolescent obesity: a case–control study in Sri Lanka

**DOI:** 10.1186/1471-2458-14-1291

**Published:** 2014-12-17

**Authors:** Kumari M Rathnayake, Tharrmini Roopasingam, VP Wickramasighe

**Affiliations:** Department of Applied Nutrition, Faculty of Livestock, Fisheries & Nutrition, Wayamba University of Sri Lanka, Makandura, 60170 Sri Lanka; Department of Paediatrics, University of Colombo, Colombo, Sri Lanka

**Keywords:** Determinants of obesity, Adolescent obesity, Sri Lankan adolescents

## Abstract

**Background:**

Global prevalence of adolescent obesity is rising at an alarming rate leading to increase risk of adult obesity. Obesity in adolescence is postulated to have a significant impact on both physical and psychological health of an individual. The study aim was to identify nutritional and behavioral risk factors associated with obesity among adolescent Sri Lankan school girls.

**Methods:**

In this case–control study, age and ethnicity matched 100 cases (BMI-for-age above +2SD) and 100 controls (BMI-for-age between -2SD to +1 SD) adolescent girls between 14 to 18 years of age were recruited. Predicted risk factors of obesity were assessed through an interviewer administrated questionnaire. A three day diet diary and long version of international physical activity questionnaire were used to assess daily energy intake and energy expenditure from physical activity, respectively. The significant differences in mean values were evaluated using paired t-test. Multivariable logistic regression analysis was performed to assess the risk factors associated with obesity.

**Results:**

Obese girls had significantly higher BMI (31.3, 20.2 kgm^−2^ p < 0.0001), waist circumference (90.8, 68.2 cm p < 0.0001), energy intake (2235.4, 1921.7 kcal p < 0.0001) and lower energy expenditure from physical activity (894.6, 1844.3 MET (metabolic equivalent)-min/week p < 0.0001). High family income (Odds ratio [OR], 2.99, 95% confidence interval [CI] 1.13-7.88), first born in family (2.73, 1.25-5.97), skipping breakfast (3.99, 1.81-8.80), consumption of fruits < 4 days per week (2.18, 1.02-4.67), screen viewing > 2 hours/ day (2.96, 1.33-6.61), energy intake (3.97, 3.19-16.36), significantly increased the risk of obesity, whereas increased physical activity (4.34, 1.33-14.14) decreased the risk. Irregular menstruation (4.34, 1.33-14.14) was noted among the obese.

**Conclusion:**

Socioeconomic and behavior factors are major determinants of adolescent obesity in Sri Lanka. There is an urgent need to implement awareness as well as behavior modification programmes targeting adolescents, parents and schools to control childhood and adolescent obesity.

## Background

Obesity in adolescence is postulated to have a significant impact on both physical and psychological health of an individual. It is an antecedent of adult obesity. The higher prevalence of childhood obesity were usually observed in developed countries. However, in recent years its prevalence has increased in developing countries such as Sri Lanka due to the socio-economic transition [1–3].

Increase in obesity over recent years is mainly due to environment and lifestyle influences rather than genetic determinants [2]. Childhood and adolescence are pivotal periods of life, where major physiological and psychological changes take place, which would transform into adult behaviour and health status. Obesity is associated with metabolic risk including high blood pressure, hypercholesterolemia, hypertriglycerinemia and insulin resistance in both old and young [4].

Adolescence is identified as a critical period for the development of obesity related metabolic derangements [5]. Majority of the studies have reported females to be at a higher risk of developing obesity and its consequences. Fat deposition begins to occur early in females with the onset of puberty and continues unless consciously controlled [6]. Thus steps should be taken to prevent obesity as early as possible in adolescence. Hence, the environmental and behaviour determinants of obesity need to be identified to design effective interventions to control this condition effectively. Recent data have shown that obesity related metabolic abnormalities are seen among Sri Lankan children of 5–10 years of age [7].

Although studies have identified adverse consequences of obesity [7], research done to identify specific risk factors associated with obesity is limited in adolescent age group. Therefore, the aim of our present study was to determine the nutritional and behavioural risk factors associated with obesity in Sri Lankan adolescent girls. This case–control study would be useful in designing effective interventions to combat adolescent obesity.

## Methods

### Study design and participants

This was a case control study of 100 subjects in each arm among pubertal adolescent school girls aged between 14 and 18 years, to examine a broad range of possible determinants of adolescent obesity. The cases (here after referred ‘obese’) and controls (here after referred ‘non-obese’) were recruited from five different schools and segregated according to BMI. BMI–for–age above +2SD and between -2SD and +1SD, of WHO 2007 growth standards, were considered as cases and controls, respectively [8]. Those who had a BMI between +1SD and +2SD were excluded. Age and ethnicity matched cases and controls were recruited on a 1:1 ratio individually from the same school and class. Ethical aspects of the study was evaluated and the Department of Applied Nutrition, Wayamba University of Sri Lanka approved the study protocol. Informed consent was obtained from the parents and assent from children before the data was collected.

### Data collection

An interviewer administered questionnaire was used to collect the information from the subjects. The questionnaire was designed to capture the dietary, behavioural and socioeconomic factors associated with obesity, from the individuals. Pubertal characteristics such as age of menarche and menstrual pattern (either regular/irregular) were obtained from the subjects during the interview.

### Dietary assessment

Information on meal frequency, patterns of meal skipping, frequency of consumption of fruit and vegetables were gathered. A 3-day diet diary, which included two weekdays and one weekend day, was used to determine the meal pattern and the nutrient intake. Subjects were asked to record all the food and drinks consumed during the day. Information on their dietary intake was taken in household measurements and was converted to grams by using standard reference tables. Mean total energy intake was calculated using FoodBase 2000 version 2, computerized food composition tables comprising nutrient compositions of Sri Lankan foods.

In addition, frequency of eating out of home, screen viewing time per day and fast food consumption patterns were obtained to assess the behaviour of the subjects. A semi quantitative food frequency questionnaire, which included common fast foods eaten by the children, was used to assess the frequency of fast food intake.

### Physical activity pattern

Total physical activity pattern for a typical week was assessed using the long version of the international physical activity questionnaire (IPAQ) [9]. It includes three types of activities; vigorous, moderate and time spent for walking. The total amount of energy spent for physical activity was calculated by multiplying the total time spent in each type of activity by the respective MET (metabolic equivalent) values and then adding up these values. Subjects were categorized into three groups; physically less active (<1500 MET-min/week), moderately active (1500–3000 MET-min/week) and highly active (≥3000 MET-min/week) based on the IPAQ cut- off values.

### Anthropometry

Body weight, height and waist circumference were measured within the school premises, in an isolated area which did not affected the routing daily activity of the school and secured the privacy of the participants, by a trained female investigators using standard equipment and guidelines [10]. Subjects were asked to remove shoes and empty their pockets, before body weight was measured using calibrated electronic scale placed on an even concrete floor (Seca 813, Hamburg, Germany) accurate to the nearest 0.1 kg. Height was measured to the nearest 0.1 cm with an upright plastic portable stadiometer (Invicta Plastics-Model IP0955, Leicester, UK). Waist circumference was measured midway between the highest point of iliac crest and the lower point of costal margin in the mid axillary line, at the end of normal expiration, using a plastic flexible tape to the nearest 0.1 cm with the subject having minimum clothing at the waist area. BMI was calculated as weight in kilograms divided by height squared in meters (kg/m^2^).

### Data analysis

The statistical analysis was carried out by using the SPSS 16.0 software. Single and multiple logistic regression analysis were performed to analyse the association between potential categorical factors. Paired t-test was performed for the continuous data to find out any significant difference between the mean values of matched cases and controls. The Odds ratio (OR) and its 95% confidence interval (CI) were also estimated and computed for each significant categorical factor using binary logistic regression. A factor with an OR significantly (p < 0.05) higher than 1.00 was taken as a risk factor of obesity, while OR significantly (p < 0.05) less than 1.00 was regarded as a protective factor. In the paired t-test analysis a factor with a p value < 0.05 was considered as significant and > 0.05 was considered as insignificant. The goodness of fit of the final logistic regression model was analysed using the Hosmer-Lemeshow technique, in which a p value >0.05 indicates a good model.

## Results

The study population consisted of 100 adolescent girls, aged between 14–18 years, in each group (obese and non-obese). Table 1 shows the general characteristics of the study sample. There were no significant differences in age, birth weight and age of menarche.

**Table 1:** **General characteristic of obese and non-obese adolescent girls**

Characteristic	Obese		Non-obese		p-value
	Mean	± SD	Mean	± SD	
Age (years)	15.34	1.29	15.33	1.29	0.957
Weight (kg)	77.5	8.5	49.18	3.5	<0.0001
Height (cm)	157.3	6.1	156. 2	4.3	0.143
BMI (kgm^−2^)	31.3	2.7	20.2	0.9	<0.0001
Waist circumference (cm)	90.8	5.3	68.2	3.7	<0.0001
Birth weight (kg)	2.81	0.57	2.92	0.45	0.129
Age at menarche (years)	11.9	1.4	12.2	1.2	0.073
Energy intake (kcal)	2235.4	252.6	1921.7	223.7	<0.0001
Total energy expenditure (MET-min/week) from physical activity	894.6	730.6	1844.3	996.3	<0.0001

P value < 0.05 was considered as significant.

Obese children had significantly higher BMI and waist circumference. The total energy expenditure from physical activity was significantly higher among the non-obese. As described in the literature, variables such as family income, mothers’ educational level, fathers’ educational level, first born status, skipping breakfast, frequency of fruit, vegetable and fast food consumption, screen viewing time, energy intake, energy expenditure from physical activity and menstruation pattern were considered as variables for logistic regression analysis. Factors which indicated significant relationship with obesity were carried forwarded for the multiple regression (Table 2).

**Table 2:** **Potential risk factors associated with adolescent obesity**

Factor	Univariable analysis		Multivariable analysis		
	OR	P value	OR	P value	95% Confidence interval ^a^
Monthly income	3.42	0.001	**2.99**	**0.027**	**1.13-7.88**
Firstborn status	3.17	0.0001	**2.73**	**0.012**	**1.25-5.97**
Skipping breakfast	3.19	0.0001	**3.99**	**0.001**	**1.81-8.80**
Fruit consumption frequency (<4 days/week)	2.93	0.0002	**2.18**	**0.045**	**1.02-4.67**
Fast food consumption frequency (≥4 days/week)	1.88	0.038	0.88	0.756	0.39-2.00
Screen viewing hours/day (>2 hours/day)	3.34	0.0002	**2.96**	**0.008**	**1.33-6.61**
Energy intake	6.73	0.0001	**7.23**	**0.0001**	**3.19-16.36**
Energy expenditure from physical activity	2.87	0.018	**3.94**	**0.012**	**1.34-11.56**
Irregular menstruation	4.47	0.001	**4.34**	**0.015**	**1.33-14.14**

OR, Odds ratio.

^a^The confidence intervals, p values and OR were obtained from corresponding multivariable binary logistic regression analysis.

Significant p values in multivariable binary logistic regression analysis (<0.05) are given in bold type.

Income of the family, being the first-born child in the family, skipping breakfast, frequency of fruits consumption, frequency of fast food consumption, screen viewing time, irregular menstruation, high energy intake from food and low energy expenditure from physical activity were associated with obesity in this group of adolescent school girls. According to multiple logistic regression analysis, the adolescent child has higher chances of being obese, when she was from a high income family and being the first child in the family. A number of behavioural risk factors have been postulated, with increasing likelihood of being obese including eating patterns, diets with a high energy density, high level of sedentary behaviour and low level of physical activity. Irregular menstruation, which is a complication of obesity, was higher among obese adolescent girls (Table 2).

## Discussion

Recent research has given much attention in identifying risk factors for obesity. Despite the existence of undernutrition and micronutrient deficiencies, recent socioeconomic transition and changes in the physical activity pattern, have led to the emergence of obesity related metabolic problems among Sri Lankan children. A recent study reported that prevalence of overweight, obesity and central obesity among Sri Lankan adults to be 25.2%, 9.2% and 26.2%, respectively with a clear upward trend [11]. Age-adjusted prevalence of Metabolic Syndrome among Sri Lankan adults was 24.3% (95% CI: 23.0–25.6) [12]. Risk factors associated with obesity in specific groups need to be identified to combat the increasing prevalence of childhood obesity. An early adiposity rebound is associated with an increased risk of adult obesity independent of parent obesity [13]. Foetal life, the period at which adiposity rebound occurs (age 4–7 y) and adolescence have been identified as pivotal periods in the development of obesity [5]. Moreover, Katulanda *et al*. have reported that female gender, urban living, higher level of education, higher income and middle age are risk factors for obesity among Sri Lankan adults [14].

Therefore, this study was carried out to identify the nutrition and behavioural risk factors associated with obesity among adolescent school girls in Sri Lanka. About, one-fifth of the total Sri Lankan population (20.4 million) consists of adolescents between the ages of 10 to 19 years [15]. Traditionally, pre and primary school children were identified as nutritionally vulnerable populations in Sri Lanka. However, at present it is quite evident that adolescent age group need more attention if we are serious about controlling non-communicable diseases among Sri Lankan adults. Most of the modifiable risk factors in obese individuals are in an adverse state. Therefore, it shows that it is important to take steps to prevent them occurring in children. This study showed that the obese individuals had a lower mean birth weight compared to the lean individuals although it was not statistically significant. Moreover, prevalence of low birth weight was higher among obese girls. This is keeping with the misconceive notion of that low birth weight babies need catch up growth, which quite eloquently shows in this study that is should not be the case. Barkers described that low birth weight was associated with non-communicable disease later in life. These relationships were independent of the Childs’ gestational age at birth and current social class [16]. Although not significant, obese girls attained menarche at a younger age than their lean counterparts. This is keeping in with the normal phenomena of obesity leading to early maturation [17]. Obese girls had significantly higher intake of energy, carbohydrate and fat compared to non-obese girls (data not shown).

A nationally representative cross-sectional study identified that skipping breakfast, inadequate consumption of green leafy vegetables and fruits, physical inactivity and increasing television viewing as potential challenges faced by Sri Lankan adolescents [18]. This study has shown the implications of those ill adoptive behaviours. Our data showed that adolescent girls of high income families were at a higher risk of being obese than those from low income families. This is the normal trend seen in economies in transition as people of affluent strata have more purchasing power to buy refined, calorie dense food in contrast to developed nations where fruits and vegetables are more expensive and high sugar and fat containing foods are available at a lower price. [19]. Keeping with the results of the present study, high prevalence of overweight was seen among adolescent girls of higher socio-economic group in urban India [20].

Our findings show an influence of birth order on obesity. It is interesting to note that the first-born status of the subject was both individually and collectively associated with higher risk of obesity at adolescent age. Previous findings suggest that although first-born offspring are lighter at birth, has increased fat mass as adolescence [21]. The mechanisms that operate are not known, but research postulates that resetting of the leptin and glucocorticoid axis within the adipocyte, contributing to increased adipo-genesis during late gestation and continuing after birth could be the mechanism [22]. It is highlighted that this may contribute to the obesity epidemic in communities where there are restrictions in family size and a generation with greater proportion of first-born children [23].

This study emphasizes the importance of targeting the family environment for the promotion of healthy eating behaviours among adolescents. Therefore, future studies should aim at identifying factors that influence feeding behaviour and the critical window at which these changes begin to occur. Even though, intake of fast food was not significantly associated with obesity in multivariable analysis, bakery items (wheat flour based food), soft drinks and candies were the most frequent fast foods consumed as snacks on a daily basis and obese adolescents consumed more fast foods than their lean counterparts. A recent study in Sri Lanka reported that unhealthy food habits are rising among adolescents, which is a crucial period in establishing dietary habits that are likely to persist into adulthood [24]. As this study shows that high energy intake and low energy expenditure (low physical activity) is associated with obesity. Therefore it is important to encourage consumption of low energy containing foods and have an active lifestyle to balance the energy.

Framingham Children’s longitudinal study showed that children who watched more television during childhood had the greatest increase in body fat over time [25]. A study conducted among adolescent school children in India revealed that the risk of overweight was seven times higher among those who had screen time ≥ 4 hours/day [26]. Our study, showed that >2 hours of screen time per day had three times higher risk of becoming obese. Furthermore, those with low physical activity had four times higher risk of being obese. Participation in physical activity programmes or sports activities during school time were less among both obese and non-obese groups. Studies have shown that adolescent participation in school physical education programs is considerably low and decreased with age. However those who engage in daily physical activity either at school or community recreation centre were associated with an increased likelihood of engaging in moderate to high physical activity during normal life [27]. High prevalence of irregular menstruation among obese girls in this study group shows that the endocrine effects of obesity begins at an early stage of life.

Some limitations of the study must be noted in the interpretation of the findings. The sample size could have been expanded more, so that stronger association of risk factors could have been demonstrated. Sine this is a case–control study, certain risk factors would have been reversed during the time of data collection as the condition is known. Therefore, the true relationship of certain current factors could have been affected.

## Conclusion

The current case control study indicates socio-economic status, firstborn status, skipping breakfast, low fruits and vegetable consumption, high screen viewing, high energy intake and physical inactivity are contributory factors for adolescent obesity. Combined adolescent and parent-focused public health interventions should be considered in addition to school programmes in order to reduce the future burden of obesity associated chronic diseases. Schools would be the best place to have prevention programmes for adolescents. However, more robust studies need to be designed to identify factors associated with adolescent obesity, in order to design better control programmes in a Sri Lankan context.
